# Inhibition of STAT3-interacting protein 1 (STATIP1) promotes STAT3 transcriptional up-regulation and imatinib mesylate resistance in the chronic myeloid leukemia

**DOI:** 10.1186/1471-2407-14-866

**Published:** 2014-11-23

**Authors:** André L Mencalha, Stephany Corrêa, Daniela Salles, Bárbara Du Rocher, Marcelo F Santiago, Eliana Abdelhay

**Affiliations:** Bone Marrow Transplantation Unit (CEMO), National Cancer Institute (INCA), Rio de Janeiro, Brazil; Biophysics and Biometry Department, Roberto Alcântara Gomes Biology Institute, Rio de Janeiro’s State University (UERJ), Rio de Janeiro, Brazil; Department of Obstetrics and Gynecology, University of Ulm, Prittwitzstrasse 43, Ulm, D-89075 Germany; Institute of Biophysics Carlos Chagas Filho (IBCCF), Federal University of Rio de Janeiro (UFRJ), Rio de Janeiro, Brazil; Departamento de Biofísica e Biometria, Instituto de Biologia Roberto Alcântara Gomes, Universidade do Estado do Rio de Janeiro, 28 Avenida de Setembro, 87 Fundos – 4 Andar, Vila Isabel, Rio de Janeiro, 20551-030 Brazil

**Keywords:** STAT3, Chronic myeloid leukemia, BCR-ABL, STATIP1, Imatinib mesylate

## Abstract

**Background:**

Signal transducer and activator of transcription 3 (STAT3) is an important transcriptional factor frequently associated with the proliferation and survival of a large number of distinct cancer types. However, the signaling pathways and mechanisms that regulate STAT3 activation remain to be elucidated.

**Methods:**

In this study we took advantage of existing cellular models for chronic myeloid leukemia resistance, western blot, *in vitro* signaling, real time PCR, flow cytometry approaches for cell cycle and apoptosis evaluation and siRNA assay in order to investigate the possible relationship between STATIP1, STAT3 and CML resistance.

**Results:**

Here, we report the characterization of STAT3 protein regulation by STAT3-interacting protein (STATIP1) in the leukemia cell line K562, which demonstrates constitutive BCR-ABL TK activity. K562 cells exhibit high levels of phosphorylated STAT3 accumulated in the nucleus and enhanced BCR-ABL-dependent STAT3 transcriptional activity. Moreover, we demonstrate that STATIP1 is not involved in either BCR-ABL or STAT3 signaling but that STATIP1 is involved in the down-regulation of STAT3 transcription levels; STATIP1-depleted K562 cells display increased proliferation and increased levels of the anti-apoptosis STAT3 target genes *CCND1* and *BCL-XL*, respectively. Furthermore, we demonstrated that Lucena, an Imatinib (IM)-resistant cell line, exhibits lower *STATIP1* mRNA levels and undergoes apoptosis/cell cycle arrest in response to STAT3 inhibition together with IM treatment. We provide evidence that STATIP1 siRNA could confer therapy resistance in the K562 cells. Moreover, analysis of CML patients showed an inverse expression of *STAIP1* and *STAT3* mRNA levels, ratifying that IM-resistant patients present low *STATIP1*/high *STAT3* mRNA levels.

**Conclusions:**

Our data suggest that STATIP1 may be a negative regulator of STAT3 and demonstrate its involvement in IM therapy resistance in CML.

## Background

The signal transducer and activator of transcription 3 (STAT3) protein belongs to a class of transcription factors that are activated by a number of growth factors and oncogenic proteins [1]. The activation of STAT3, which is regulated by the phosphorylation of tyrosine 705, is driven by receptor and non-receptor protein tyrosine kinases (TK), such as EGFR, gp130, Ras, Src and Abl [2–5]. Once activated, STAT3 forms homodimers, translocate to the cell nucleus and binds to specific regulatory DNA elements to induce transcription. Under physiologic conditions, the activation of STAT3 is transient and rapid [6]. However, the persistent activation of STAT3 protein has been associated with several hematological cancers and solid tumors [7]. Previous data suggest that the constitutive activation of STAT3 induces cell transformation by the up-regulation of anti-apoptotic and cell proliferation-related genes, such as *BCL*-*XL* and *CCND1* [7], and oncogenes, such as *PIM1* and c-Myc [8, 9]. Furthermore, STAT3 activation has been associated with the up-regulation of *VEGF* and *TWIST1*, genes related to angiogenesis and metastasis [10]. These findings suggest a straight relationship between STAT3 activation and cancer development.

In chronic myeloid leukemia (CML), the chimeric oncoprotein BCR-ABL, a constitutively activated TK, promotes the malignant transformation of hematopoietic cells [11]. BCR-ABL leads to the constitutive activation of the JAK/STAT, Ras/Raf/MEK/ERK and PI3K/PTEN/Akt/mTOR signaling pathways [12–14]. In CML, persistent STAT3 phosphorylation mediated by BCR-ABL has been associated with cellular proliferation, the inhibition of apoptosis and chemotherapy resistance [5, 15–19]. Although it is clear that the signaling activity of BCR-ABL is the main cause of the neoplastic transformation, the precise mechanisms by which BCR-ABL transforms cells remain largely unknown. Thus, strategies designed to understand the transcriptional activity of STAT3 may be important tools for discovering the next generation of anti-leukemia therapies.

STAT3 is negatively regulated by the suppressors of cytokine signaling proteins, known as SOCS, by protein inhibitor of activated STAT, known as PIAS, or by phosphatases, known as SHP. However, the regulatory mechanisms that negatively modulate STAT3 are ineffective in cancers [20]. Thus, several studies have tried to identify proteins that could interact and positively or negatively regulate STAT3 activity [21–28].

Although many proteins are known to interact and regulate STAT3 activity, the mechanisms surrounding such regulation of the STAT3 protein remain to be elucidated in CML. Collum and cols. [29] described STAT3-interacting protein 1 (STATIP1) as a STAT3-associated protein. STATIP1 contains 12 WD40 domains that mediate protein-protein interactions, which play important roles in the regulation of signal transduction, transcription and proteolysis [30]. STATIP1 overexpression blocked STAT3 activation in the human hepatocellular carcinoma cell line HepG2 [29], suggesting a negative role for STATIP1 in STAT3 regulation. However, neither the STATIP1 expression nor its potential to regulate STAT3 activity has been assessed to date in other cancer types, such as leukemia cells. To address this issue, the aim of this study was to evaluate the STATIP1 and STAT3 status in the well-characterized CML model. Using K562 cell line, we report that STATIP1 may act as a negative regulator of STAT3 transcriptional activity in CML and reduce the effects of Imatinib (IM) in K562 cells. Moreover, using a CML multidrug resistance (MDR)/Imatinib resistant cell line (Lucena) and CML patients’ samples we address the relationship of STATIP1 and STAT3 in IM resistance. Our results suggest a new role for STATIP1 in CML therapeutic resistance.

## Methods

### Cell lines and drug treatments

A CML model cell line, K562, was cultured in RPMI-1640 medium containing 10% fetal bovine serum, 100 U/ml penicillin and 100 μg/ml streptomycin in 5% CO2 at 37°C. Lucena cells [K562 MDR/IM resistant cell line induced by vincristine] overexpressing *ABCB1* were kindly provided by Dra. Vivian Rumjanek (Departamento de Bioquímica Médica, Universidade Federal do Rio de Janeiro, Brazil) [31]. The Lucena cells were cultured in the same conditions as the K562 cells, but its medium was supplemented with 60 nM VCR (Sigma).The K562 cells were plated at 1 × 10^5^ cells/ml. The inhibition of BCR-ABL activity by treatment with IM (imatinib mesylate, Novartis) was performed using a final concentration of 1 μM for 24 h. For STAT3 inhibition, 40 μM LLL-3 was applied to culture for 24 h. The LLL-3 was kindly provided by Dr. Pui-Kai Li from Ohio State University, USA.

### Patients samples

This study was approved by the ethics committee of the National Cancer Institute Hospital (INCA, Rio de Janeiro, Brazil). Patients were admitted or registered at the National Cancer Institute Hospital, according to the guidelines of its Ethics Committee and the Helsinki declaration. All patients and healthy donors were adults and signed the consent form. Bone marrow samples were obtained from CML patients in all disease phases (chronic, accelerated and blastic phases) at the time of diagnose and follow up: IM-responsive patients (3 to 6 mo follow up) and IM-resistant or relapse after initial response (3 to 24 mo follow up). We selected 6 healthy donors (mean age =30, range =20-37, male:female ratio = 4:2), 6 IM-responsive patients (mean age = 45, range = 35–68, male:female ratio = 1:5) and 8 IM-resistant patients (mean age = 51, range = 24–59, male: female ratio = 6:2). Diagnoses and follow-ups were based on hematologic, cytogenetic and molecular assays. IM-responsive patients exhibited a major molecular response and complete hematologic and cytogenetic response, whereas IM-resistant patients lacked hematologic, cytogenetic and molecular responses. The inclusion criterion was to investigate CML patients that received IM as a first-line therapy. The exclusion criterion was CML patients with BCR-ABL mutations. Marrow aspirates were collected in heparinized tubes and processed on the day they were collected. Bone marrow mononuclear cells were isolated from 2–5 mL of aspirate in a Ficoll-Hypaque density gradient (Ficoll 1.077 g/mL; GE, Sweden) according to manufacturer’s protocol. Cells were washed 3 times in PBS and subsequently used for RNA extraction.

### Small interfering RNA (siRNA)

TK562 cells were plated at 1 × 10^5^ cell/ml in a 24-well plate and left overnight in RPMI-1640 media without antibiotics. STATIP1 siRNA (100 nM) (SC-44436, Santa Cruz) and 2 μL of Lipofectamine™ RNAiMAX (Invitrogen) were incubated separately in a final volume of 50 μL of RPMI-1640 media for 5 min. Subsequently, the siRNA and Lipofectamine were mixed and incubated for 30 min and then applied dropwise on cell cultures. Scrambled siRNA (100 nM) (SC-37007, Santa Cruz) was used as an siRNA negative control. FITC-conjugated siRNA (SC-36869, Santa Cruz) was used to evaluate the transfection efficiency by FACS. siRNA transfections were conducted for up to 72 h.

### Proliferation assay

K562 cells (1 × 10^5^) were transfected with scrambled or STATIP1 siRNA in a 24-well plate for 72 h. After transfection, cell cultures were treated with 1 μM IM for 24 h. WST-1 assay was performed to determine the number of viable cells. The relative number of viable cells was expressed as a percentage of the untreated cells.

### Real time quantitative PCR (RT-qPCR)

Total RNA was extracted from IM-treated and untreated cells using TRIzol reagent (Invitrogen). Total RNA was subjected to treatment with a DNAse Amplification Grade I Kit (Invitrogen) for the removal of DNA contamination. Complementary DNA synthesis was performed with Superscript-II Reverse Transcriptase (Invitrogen) following the manufacturer’s protocol. Quantitative Real-Time PCR (RT-qPCR) was performed with SYBR Green Master Mix (Invitrogen) in a Rotor-Gene Q (Qiagen). The following forward (Fow) and reverse (Rev) primers were used: *STAT3* - Fow 5’ GGGAGAGAGTTACAGGTTGGACAT 3’, Rev 5’ AGACGCCATTACAAGTGCCA 3’; *STATIP*1 - Fow 5’ CCACTGTCCCTGCATTGGGATT 3’, Rev 5’ GCCACCTGCTGATACTCAAA 3’; *CCND1*- Fow 5’ AGAGACCAGGCTGTGTCCCTC 3’, Rev 5’ GTGGTGGCACGTAAGACACAC 3‘; *BCL*-*XL* Fow 5’ CTGGGGTCGCATTGTGGC 3’, Rev 5’ AGCCGCCGTTCTCCTGGA 3’; *ABCB1* - Fow 5’ CCCATCATTGCAATAGCAGG 3’, Rev 5’ GTTCAAACTTCTGCTCCTGA 3’; *ACTB* - Fow 5’ ACCTGAGAACTCCACTACCCT 3’, Rev 5’ GGTCCCACCCATGTTCCAG 3’. The PCR cycling conditions included an initial denaturation of 95°C for 10 minutes, followed by 45 cycles of 20 seconds at 95°C, 20 seconds at 60°C, and 40 seconds at 72°C. The β-actin mRNA levels were used as a reference of expression. The fold-expression was calculated according to Schmittgen and Livak [32]. The primer sequences used in this work are available upon request.

### Western blot

Whole-cell protein extracts were obtained from cell lines in lysis buffer containing 50 mM Tris pH 7.5, 5 mM EDTA, 10 mM EGTA, 50 mM NaF, 20 mM b-glycerolphosphate, 250 mM NaCl, 0.1% Triton X-100, 20 mM Na_3_VO_4_ and protease inhibitor mix (Amersham). The protein concentrations were determined using the Bradford assay, and 30 μg of the cell lysate proteins was subjected to separation by 10% SDS-PAGE. The protein extracts were electrophoretically transferred to a nitrocellulose membrane (GE) and probed with the appropriate antibodies. The western blots were developed by ECL Plus (Amersham). The following antibodies were used at 1:1000 dilutions: anti-STATIP1, anti-STAT3, anti-STAT3-Y705 and anti-ACTNB (Santa Cruz).

### Immunofluorescence

K562 cells were fixed to glass slides using cytospin and further fixed by immersion in methanol:acetic acid (1:1) for 10 min at -20°C. Fixed cells were permeabilized in 0.5% Triton X-100 for 10 minutes and blocked with 5% BSA for 1 h. Primary antibody incubation was performed at 4°C for 16 h. The cell nuclei were stained with DAPI (Santa Cruz). The images were analyzed using a LSM 510 Meta (Carl Zeiss) microscope equipped with a 63×/1.4 NA Plan-Apochromat oil immersion objective.

### Apoptosis assay

To determine the percentage of apoptotic cells, we analyzed phosphatidyl serine externalization and membrane integrity by double staining with Annexin V PE and 7-AAD (PE Annexin V Apoptosis Detection Kit I, BD Pharmingen, USA) according to manufacturer's instructions. Briefly, after treatment, 1.0 × 10^5^ cells were harvested, washed twice with cold PBS and resuspended in 100 μL of 1× binding buffer. Annexin V PE (5 μL) and 7-AAD (5 μL) were added, and samples were incubated for 15 min in the dark. After incubation, 400 μL of 1X binding buffer was added to each sample. Cells positive for Annexin V PE and 7-AAD were considered apoptotic. For every condition, 20.000 events were acquired using a FACSCalibur Flow Cytometer (Becton Dickinson, USA) and analyzed using CellQuest v.3.1 Software (Becton Dickinson, USA). All experiments were performed in triplicate.

### Cell cycle assays

Cell cycle was evaluated by staining with propidium iodide (PI, Sigma-Aldrich) [33]. Approximately 3.0 × 10^5^ cells were resuspended in 400 μL of hypotonic buffer (3.4 mM Tris-HCl (pH 7.6), 10 mM NaCl, 0.1% (v/v) NP-40, 700 U/L RNase, and 0.075 mM PI) and incubated for 30 min at 4°C. For every condition, 5.000 events were acquired in a FACSCalibur Flow Cytometer (Becton Dickinson, USA) and analyzed using Cell Quest v.3.1 Software (Becton Dickinson, USA). All experiments were performed in triplicate.

### Statistical analysis

All of the experiments were repeated at least three times, and the data are expressed as the mean ± SD. Statistical analyses (ANOVA and t-test) were performed using GraphPad Prism® v.5 software (GraphPad). A P-value (p) <0.05 was considered statistically significant (*p <0.05, **p <0.01, ***p <0.001).

## Results

### Evaluation of STAT3 expression and phosphorylation in CML K562 cells

**Figure 1:**
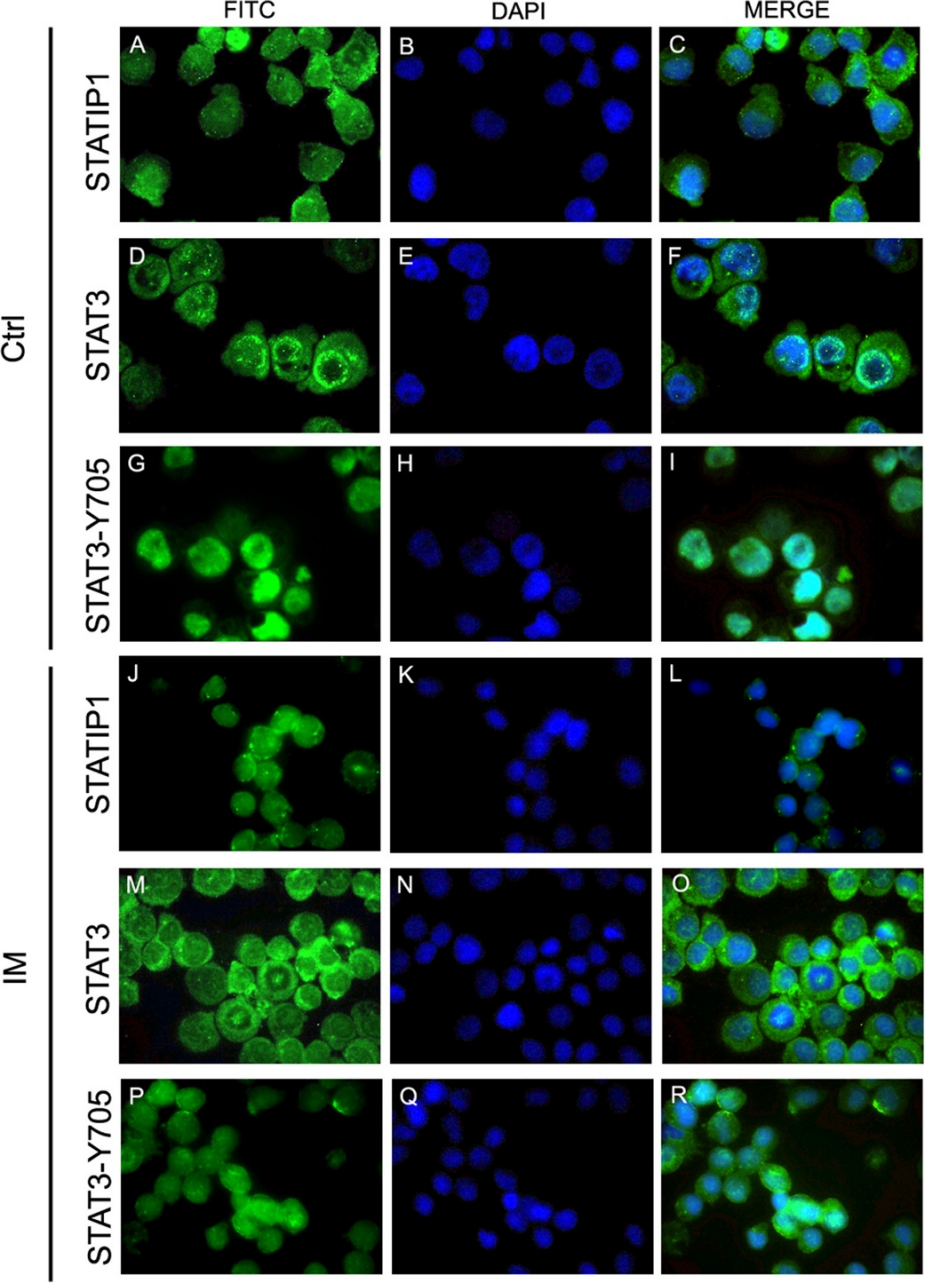
**Immunofluorescence analyses of STAT3, STAT3-Y and STATIP1 proteins.** STAT3, STAT3-Y and STATIP1 FITC-labeled antibodies (green), DAPI-stained DNA (blue) and merged images. Protein labeling was observed in untreated K562 cells **(A-I)** and K562 cells treated with 1 μM IM **(J-R)**. The slides were analyzed using an LMS confocal system, and the images were processed using AxioVision-LE software (Carl Zeiss).

Previous studies have demonstrated that STAT3 is constitutively activated in a variety of cancer cell types [7], including leukemic cells [34]. First, we evaluated the STAT3 expression and phosphorylation status and sub-cellular localization in our CML cell line, K562. For this, immunofluorescence assays and western blot analyses were performed. Our results indicate that STAT3 is preferentially localized in the K562 cytoplasm, while a very strong nuclear accumulation of phosphorylated STAT3 is observed in these cells (Figure 1F, 1I). These findings indicate that when STAT3 is phosphorylated, it accumulates in the K562 cell nucleus. These data validate our model as a STAT3-activated leukemic cell line, as reported by Benekli and cols. [7], who described STAT3 phosphorylation as a common finding in leukemic and other cancer cells.

### Inhibition of BCR-ABL interferes with STAT3 modifications but does not alter STATIP1 protein expression

**Figure 2:**
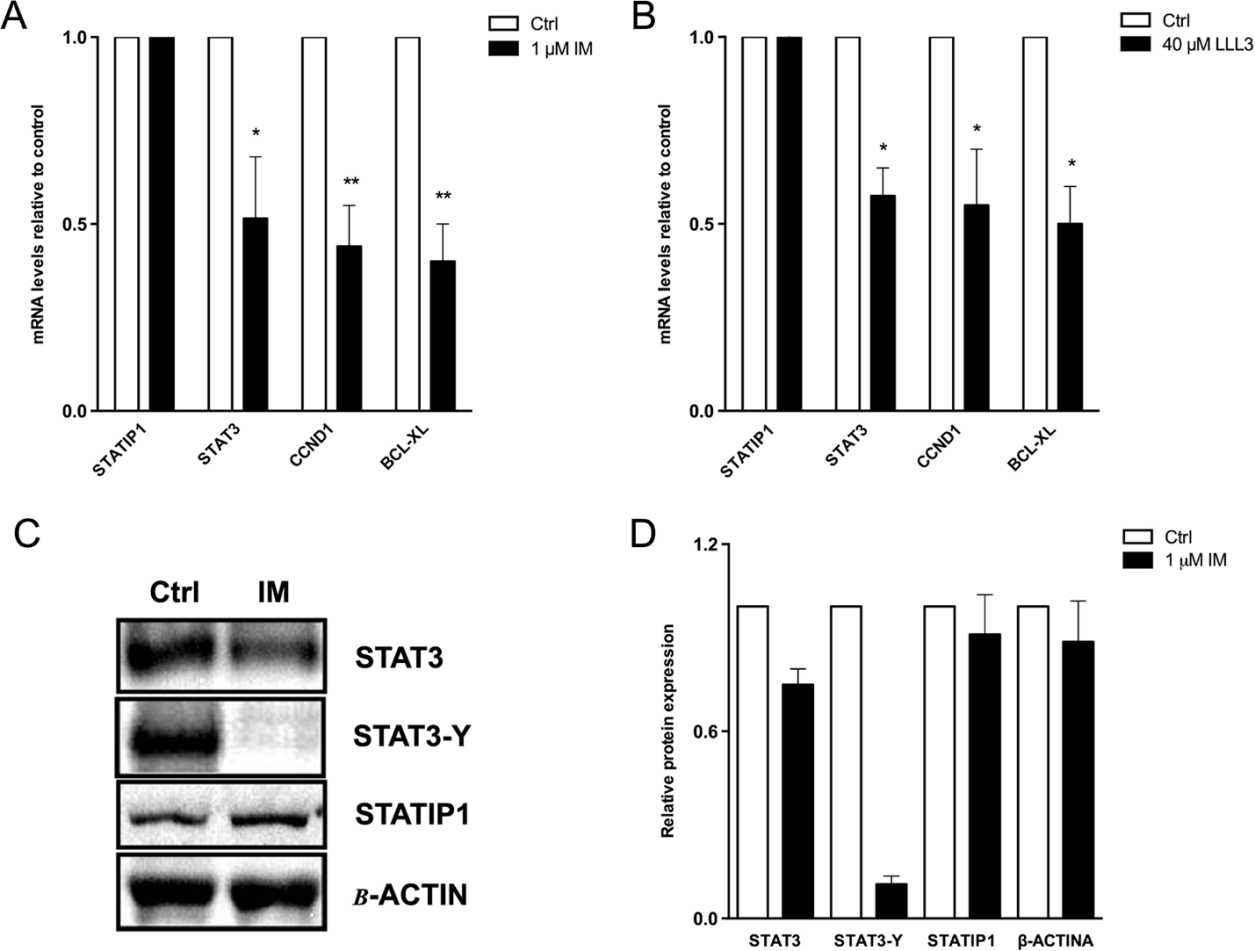
**Expression levels of** ***STATIP1*** **,** ***STAT3*** **,** ***CCND1*** **and** ***BCL-XL*** **genes in response to IM/LLL-3 treatments. (A)** Relative mRNA levels of *STATIP1, STAT3, CCND1* and *BCL-XL* after 24 h of 1 μM IM treatment. **(B)** Relative mRNA levels of *STATIP1, STAT3, CCND1* and *BCL-XL* after 24 h of 40 μM LLL-3 treatment. **(C)** Western blot analysis of STAT3 and STATIP1 protein levels and STAT3-Y705 phosphorylation 24 h after 1 μM IM treatment. **(D)** The protein levels were determined by densitometry analysis in ImageJ software version 1.44. All comparisons were made to untreated cells – ctrl. Ctrl: control. The data represent the mean ± SD of at least three independent experiments (*p <0.05 and **p <0.01).

To demonstrate the role of BCR-ABL in STAT3 phosphorylation and the possible consequence of this signaling on STATIP1 expression, we first investigated the status of STAT3 and STATIP1 expression and STAT3 tyrosine-705 phosphorylation in BCR-ABL-inhibited K562 cells by immunofluorescence assays and western blotting. We inhibited BCR-ABL activity with 1 μM IM (Figure 1J-R), as previously described [35]. Although BCR-ABL coordinates several molecular alterations, the STATIP1 protein levels remained unaltered following BCR-ABL inhibition using 1 μM IM for 24 h (Figure 1C, 1L). However, the STAT3 protein levels, phosphorylation status and nuclear accumulation were decreased in IM-treated cells compared with non-treated K562 cells (Figures 1R and 2A, C-D). Unlike STAT3, our data suggested that STATIP1 expression is not related to BCR-ABL signaling (Figures 1L and 2A, C-D).

### Imatinib treatment induces down-regulation of STAT3 target genes but not alteration of STATIP1 transcript levels

Several genes listed as STAT3 targets exhibit a relevant role in cancer [7–10]. STAT3 target genes mainly include cellular growth promoters and inhibitors of apoptosis [36]. Moreover, STAT3 has been described as an activator of its own transcription [37]. Here, we investigated the regulation of STAT3 target genes in K562 cells in response to IM treatment. The mRNA levels of *CCND1*, *BCL*-*XL* and *STAT3* genes were measured by RT-qPCR. Our results suggest that STAT3 target genes were down-regulated 24 h after IM treatment (Figure 2A). To assess the direct activity of STAT3 on its gene targets, we directly inhibited STAT3 using LLL-3. In corroboration with the previous results, the *CCND1*, *BCL*-*XL* and *STAT3* mRNA levels were down-regulated in K562 cells after 24 h with LLL-3 treatment compared to untreated cells (Figure 2B). These findings indicate that STAT3 inhibition either indirectly, by IM, or directly, by LLL-3, induces a decrease in STAT3 transcriptional activity. Additionally, STAT3 inhibition with LLL-3 also does not interfere with the *STATIP1* mRNA levels (Figure 2B). Our data indicated that STATIP1 is not correlated with either the BCR-ABL or STAT3 signaling pathways but that it may be related to STAT3 activity in the CML cell line.

### STATIP1 depletion results in increased STAT3 transcriptional activity in K562 cells

**Figure 3:**
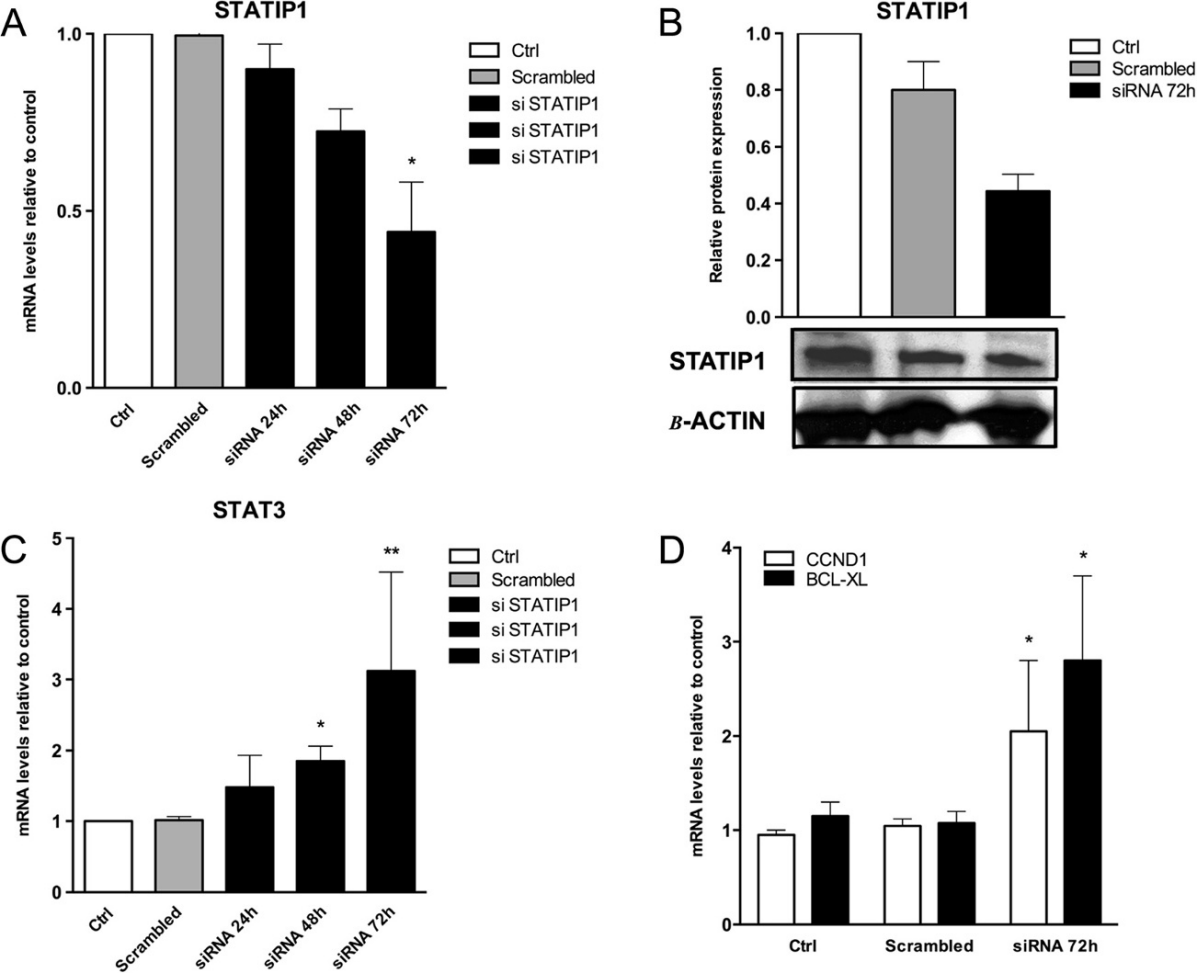
***STATIP1*** **mRNA depletion by siRNA induces the over-expression of** ***STAT3*** **and its target genes. (A)** *STATIP1* mRNA levels at 24 h, 48 h and 72 h after STATIP1 silencing, as determined by RT-qPCR. **(B)** Western blot analyses of the STATIP1 protein level 72 h after STATIP1 silencing. **(C)** RT-qPCR analyses of the *STAT3* mRNA levels at 24 h, 48 h and 72 h after STATIP1 silencing. **(D)** RT-qPCR analyses of the *CCND1* and *BCL-XL* mRNA levels at 72 h after STATIP1 silencing. All comparisons were made to untreated cells – ctrl and scrambled-treated cells. Ctrl: control. The data represent the means ± SD of at least three independent experiments (*p <0.05 and **p <0.01).

Previous studies have demonstrated that STAT3 activity can be regulated by STAT3 protein interactions [23, 27, 38]. To determine the potential of STATIP1 in regulating the transcriptional activity of STAT3, K562 cells were transfected with siRNA against STATIP1. The mRNA levels were analyzed and compared to untransfected or scrambled-transfected K562 cells. By RT-qPCR, significant decreases in the *STATIP1* mRNA and protein levels were observed 72 h after siRNA transfection (Figure 3A,B). Interestingly, the increase in *STAT3* mRNA levels after STATIP1 inhibition were inversely proportional, showing significant elevation at 72 h (Figure 3C). This result suggests that with transient STATIP1 depletion, *STAT3* is more transcriptionally activated. To validate this hypothesis, we investigated STAT3 target gene mRNA levels. Surprisingly, in response to STATIP1 inhibition, a significant two-fold increase of *CCND1* mRNA levels and a three-fold increase of *BCL*-*XL* mRNA levels were observed 72 h after siRNA transfection (Figure 3D). These findings showed that STATIP1 down-regulation in K562 cells augments the STAT3 mRNA levels and its targeted genes, demonstrating that STATIP1 is involved (directly or indirectly) in the negative regulation of STAT3 transcription.

### STATIP1 is involved in imatinib resistance in CML

**Figure 4:**
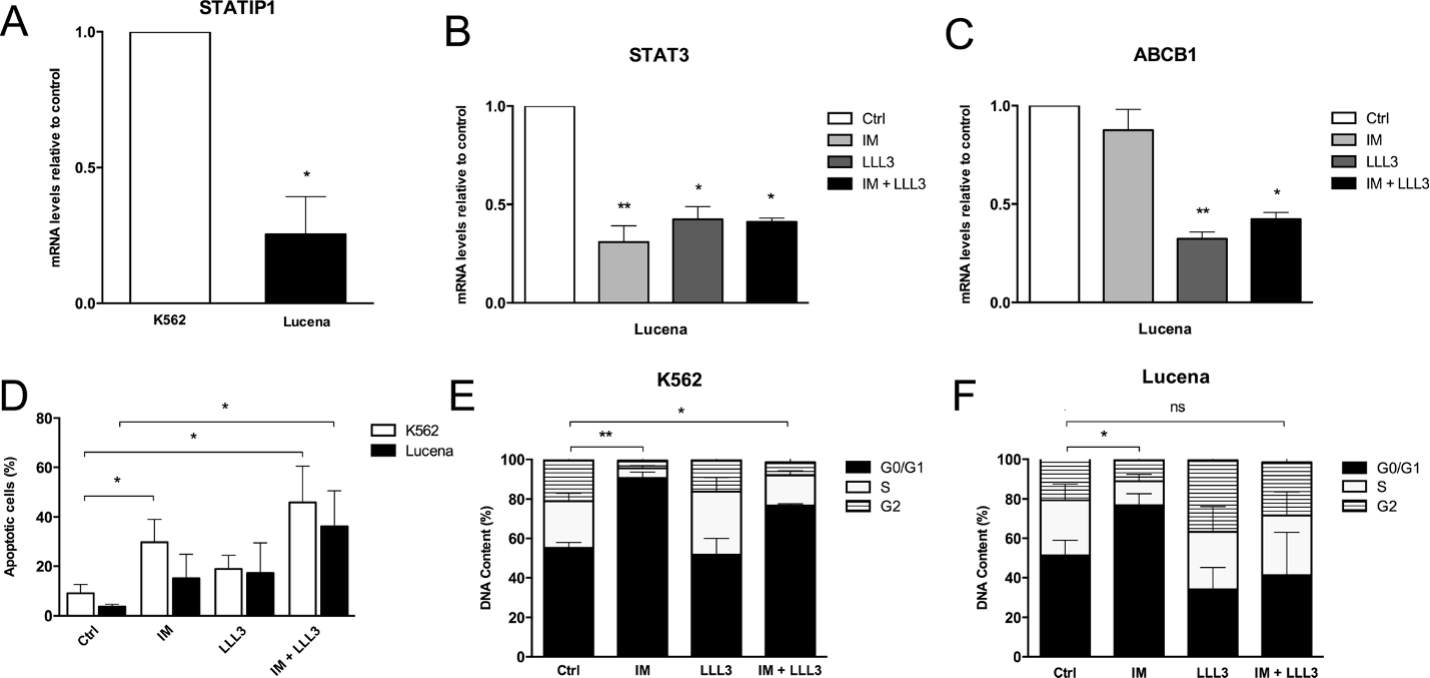
**Involvement of** ***STATIP1*** **and** ***STAT3*** **genes in IM resistance in CML cell lines. (A)** *STATIP1* mRNA levels in K562 and Lucena cells determined by RT-qPCR. **(B)** *STAT3* mRNA levels, as determined by RT-qPCR, in Lucena cells under the following conditions: 1 μM IM treatment, 40 μM LLL-3 treatment, and co-treatment after 24 h. **(C)** The *ABCB1* mRNA levels in Lucena cells were determined by RT-qPCR under the treatment conditions noted above. **(D)** Apoptotic cells were measured by flow cytometry in both cell lines under the treatment conditions noted above. The cell cycle was evaluated by flow cytometry after being subjected to the treatment conditions noted above in K562 **(E)** and Lucena **(F)** cells. All comparisons were made to untreated cells – ctrl. Ctrl: control. The data represent the mean ± SD of at least three independent experiments (*p <0.05 and **p <0.01).

The role of STATIP1 both physiologically and in cancer cells is completely unknown. In an effort to determine the mechanism of STATIP1-mediated CML therapy resistance, we used the Lucena cell line as a model of IM resistance [39]. Lucena cells were subjected to IM, LLL-3, and co-treatment (as previously reported) [35], and the *STAT3*, *STATIP1* and *ABCB1* mRNA levels were evaluated after 24 h and compared to untreated cells. The *STATIP1* mRNA levels were lower in the Lucena cells compared to untreated K562 cells (Figure 4A). Additionally, the *STAT3* mRNA levels decreased by 60% in the Lucena cells with each of the different treatments (Figure 4B), but the *ABCB1* mRNA levels only decreased with the LLL-3 treatment (≅50%) (Figure 4C). No differences were observed regarding the *ABCB1* mRNA levels in K562 cells (data not shown). Interestingly, STAT3 inhibition by LLL-3 treatment sensitized Lucena cells to IM treatment (Figure 4D) in a cell cycle arrest-independent manner (Figure 4E-F). Together, these results suggest that IM resistance may be associated not only with *STAT3* overexpression/activation but also with *STATIP1* down-regulation.

**Figure 5:**
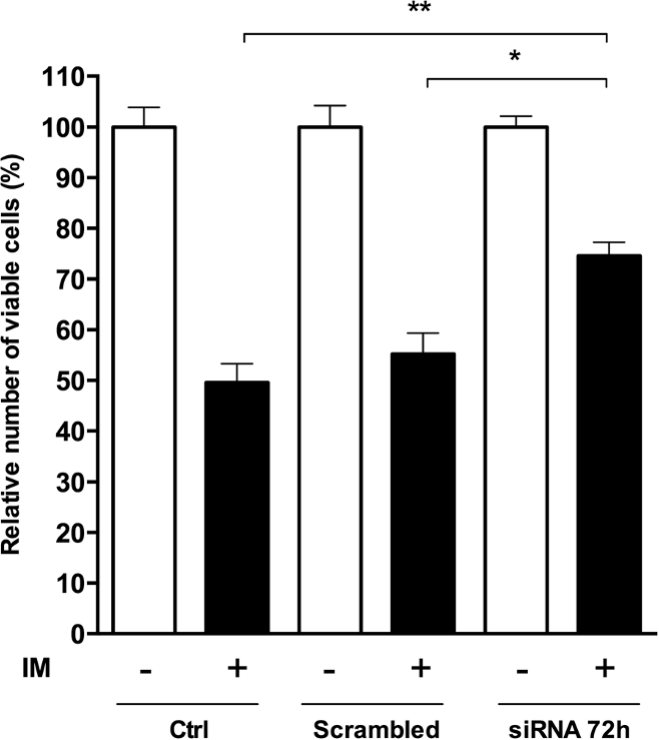
**Evaluation of cell viability in STATIP1-silenced K562 cells after IM treatment.**The relative percentage of viable cells was determined by WST-1 assay after 24 h of IM treatment (+), compared to untreated cells – ctrl and scrambled-treated cells. Ctrl: control. The data represent the mean ± SD of at least three independent experiments (*p <0.05 and **p <0.01).

**Figure 6:**
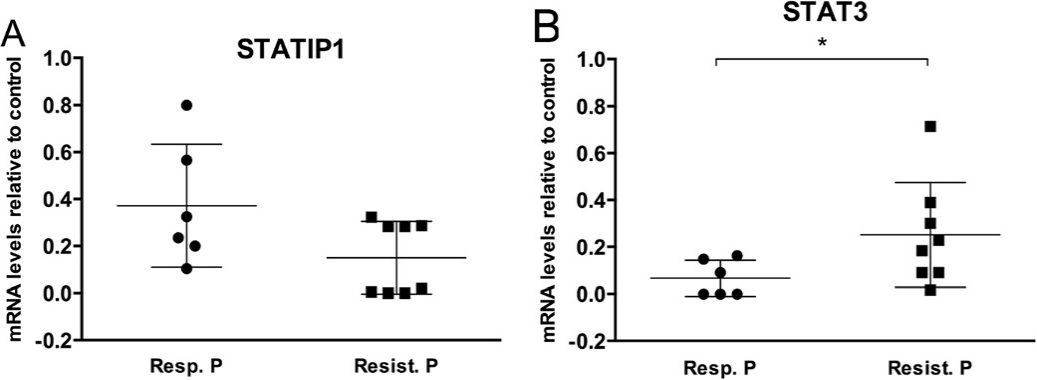
**Expression levels of** ***STATIP1*** **and** ***STAT3*** **genes in CML patients. (A)** *STATIP1* mRNA levels and **(B)** *STAT3* mRNA levels were determined by RT-qPCR analyses in 6 IM-responsive patients and 8 IM-resistant patients. Raw expression values were normalized to β-actin expression. Expression changes were calibrated by 6 healthy bone marrow donors analysis. Resp. P = responsive patients; Resist. P. = resistant patients. (*p <0.05).

To address this hypothesis, we assessed K562 cell viability after IM treatment in STATIP1-depleted cells 72 h after siRNA transfection. Our results indicated a decrease in the IM sensitivity of the K562 cell line with reduced STATIP1 expression compared to the control or scrambled K562 cells (Figure 5). After 24 h of 1 μM IM treatment, approximately 25% of the STATIP1-depleted K562 cells remained viable compared to the control or scrambled cells (Figure 5). Additionally, we also analyzed a total of 14 CML patients with different responses to IM (6 IM-responsive and 8 IM-resistant) and 6 healthy bone marrow donors. RT-qPCR analyses showed that IM-resistant patients presented *STATIP1* mRNAs levels down-regulated, compared to IM-responsive patients (Figure 6A). Moreover, *STAT3* mRNA levels were inversely expressed; up-reguleted in IM-resistant patients, compared to IM-responsive (Figure 6B). These data suggest that the decreased expression of STATIP1 may promote IM resistance in the K562 cell line, and could be an important piece of *in vivo* IM-resistance development in CML.

## Discussion

Although the BCR-ABL oncoprotein, a hallmark of CML, constitutively activates multiple signaling pathways [3], our group was particular interested in STAT3 signaling activation, as the constitutive activation of STAT3 is associated with oncogenic transformation induced by the viral Src oncoprotein [2]. Furthermore, many *in vivo* and *in vitro* assays have demonstrated the association of STAT3 activation with the development and maintenance of several cancer types [1, 4, 10, 16]. Despite the known association of STAT3 phosphorylation with cancer, the mechanisms that regulate STAT3 are not well understood.

In this study, we were able to clarify the relationship between BCR-ABL signaling and STAT3 activation. Our data indicated strong STAT3 phosphorylation and nuclear accumulation in untreated K562 cells. K562 treatment with IM, an inhibitor of BCR-ABL activity, not only promoted a decrease in the mRNA and protein levels of STAT3 but also inhibited STAT3 phosphorylation. Moreover, our results also showed a transcriptional positive feedback loop, suggesting that STAT3 promotes its own over-expression, which may be important to signaling intensification. In summary, our findings suggest that STAT3 is phosphorylated and transcriptionally activated by BCR-ABL activity in K562 cells.

Several studies have demonstrated that STAT3 signaling can regulate the expression of numerous genes that are frequently involved with proliferation and apoptosis [34], angiogenesis, metastasis and differentiation [36], some of which are capable of positively regulating STAT3 through protein-protein interactions. To probe the diminished STAT3 activation in BCR-ABL-inhibited cells, we assessed the expression of representative known STAT3 target genes involved in proliferation and cellular survival, *CCND1* and *BCL*-*XL*, respectively, and STATIP1, a protein identified in a two-hybrid assay as interacting with STAT3 [29]. As expected, *CCND1* and *BCL*-*XL* were down-regulated in response to IM treatment, but unlike *STAT3*, the *STATIP1* mRNA and protein levels were unaltered in the treated cells. Accordingly, our results indicated that STATIP1 was not affected by the molecular alterations promoted by BCR-ABL signaling.

Hawkes and cols. characterized the STATIP1 levels in the cytoplasm and nuclei of cancer cell lines exercising multiple distinct roles that are dependent on its sub-cellular localization [40]. To further investigate the relationship between STAT3 and STATIP1 in the context of BCR-ABL, we inhibited STAT3 activity with LLL-3, a more direct approach that has been previously used by our group [35]. Similar to the BCR-ABL inhibition experiments, previously investigated STAT3 target genes demonstrated decreased mRNA levels compared to untreated cells. STATIP1 remained unchanged in K562 cells treated with the STAT3 drug inhibitor LLL-3. This result corroborates our previous results, again suggesting that STATIP1 expression is not related to molecular signaling changes driven by either BCR-ABL or STAT3. Moreover, our findings showed that STATIP1 is present in both the cytoplasm and nuclei of K562 cells. Further characterization of the localized STATIP1 pools could reveal its precise role in these cellular compartments.

It is known that STATIP1 contains 12 WD40 domains that are responsible for mediating protein-protein interactions that play important roles in signal transduction regulation, transcription and proteolysis [30]. In this context, the investigation of the role of STATIP1 in signal transduction showed that its forced over-expression is able to block STAT3 activation [29]. However, the regulation of STAT3 transcriptional activity by STATIP1 was only observed in the human hepatocellular carcinoma cell line HepG2 [29]. In this study, we characterized STATIP1 in the K562 cell line and investigated its role in STAT3 transcriptional activity in a distinct cell line established from another cancer type, chronic myeloid leukemia. Instead of over-expressing STATIP1, as was performed by Collun and cols. [29], we depleted the *STATIP1* mRNA and protein levels to investigate the role of STATIP1 in regulating STAT3 transcriptional activity in K562 cells. Our results showed a gradual increase of STAT3-target gene mRNA levels, such as those of *STAT3*, *CCND1* and *BCL*-*XL*, in K562 cells subjected to STATIP1 inhibition. Similarly to Collun [29], our findings also indicated that STATIP1 may work as a negative regulator of STAT3 transcriptional activity. Because STATIP1 interacts with STAT3, we inferred that this may be a direct regulation mechanism. Indeed, existing data have already characterized STATIP1 protein as a scaffolding protein that regulates the activity of interacting proteins [40]. Based on this finding, we propose that STATIP1 may interact with STAT3 in K562 cells and regulate STAT3 activation. However, additional investigation is required to address the intricate mechanism by which STAT3 is inhibited by STATIP1. Nevertheless, independent of whether it is a direct or indirect regulation and how it works precisely, our results demonstrated that negative regulation of STAT3 by STATIP1 appears to be a common issue in distinct cancer cell types. If this result is validated in other diverse cancer cell types, we propose that STAT3 regulation may be important to cancer development and that it may also be an interesting target for the design of new drug strategies against cancer cells.

Because STAT3 over-expression is closely related to CML drug resistance and has been implicated in a poor prognosis [17, 41], we evaluated the role of STATIP1 in IM resistance. We took advantage of an IM-resistant cell model, the Lucena cell line. Lucena cells exhibit a multidrug resistance phenotype (with *ABCB1* over-expression) and have been shown to also be IM resistant, compared to K562 cells [39]. We investigated the *STATIP1*, *STAT3*, and *ABCB1* mRNA levels, together with apoptosis and cell cycle arrest, in Lucena cells with the inhibition of BCR-ABL and STAT3.

We observed decreased *STATIP1* mRNA levels in Lucena cells compared to K562 cells. Because Lucena cells are resistant to IM, we observed *STAT3* down-regulation in all of the treatments; additionally, we observed a decrease in the *ABCB1* mRNA levels. This result was expected because it is known that *ABCB1* is a STAT3 target [42, 43]. Moreover, STAT3 direct inhibition (LLL-3 treatment) induced Lucena cells to undergo apoptosis, in contrast to indirect inhibition (IM treatment), and this effect was independent of cell cycle arrest. This result demonstrated that STAT3 over-expression together with STATIP1 down-regulation could be involved in IM resistance.

To validate this hypothesis, we depleted STATIP1 and inhibited BCR-ABL activity in K562 cells and assessed the proliferation and survival. Interestingly, our results demonstrated that STATIP1-depleted K562 cells have a higher survival percentage than control or scrambled-transfected cells. STAT3 can overcome sensitivity to BCR-ABL inhibition by driving proliferation, anti-apoptosis and MDR gene expression, increasing CML cell survival [15–19, 43]. Moreover, althought we analyzed a small cohort of healthy donors and patients samples, our *in vivo* analyses suggested that *STAT3* and *STATIP1* genes are inversely expressed in IM-response, which corresponds to our findings in K562 and Lucena cell lines. The present study is the first report of *STATIP1* expression in CML patients with different responses to IM therapy. Further studies may reveal the details of STATIP1 role in IM resistance.

## Conclusions

Our data suggest that STATIP1 may be a negative regulator of STAT3 and that it could be involved in the acquisition of therapeutic resistance to IM in CML.

## Abbreviations

CML: Chronic myeloid leukemia
Fow: Forward
IM: Imatinib mesylate
PE: Phycoerythrin
PI: Propidium iodide
Rev: Reverse
RT-qPCR: Real-time quantitative PCR
siRNA: Small interfering RNA
TK: Tyrosine kinase.
